# GNSS Spoofing Network Monitoring Based on Differential Pseudorange

**DOI:** 10.3390/s16101771

**Published:** 2016-10-23

**Authors:** Zhenjun Zhang, Xingqun Zhan

**Affiliations:** School of Aeronautics and Astronautics, Shanghai Jiao Tong University, Shanghai 200240, China; zj.zhang@sjtu.edu.cn

**Keywords:** GNSS, spoofing and anti-spoofing, spoofing network monitoring, TDOA, differential pseudorange to carrier frequency ratio

## Abstract

Spoofing is becoming a serious threat to various Global Navigation Satellite System (GNSS) applications, especially for those that require high reliability and security such as power grid synchronization and applications related to first responders and aviation safety. Most current works on anti-spoofing focus on spoofing detection from the individual receiver side, which identifies spoofing when it is under an attack. This paper proposes a novel spoofing network monitoring (SNM) mechanism aiming to reveal the presence of spoofing within an area. Consisting of several receivers and one central processing component, it keeps detecting spoofing even when the network is not attacked. The mechanism is based on the different time difference of arrival (TDOA) properties between spoofing and authentic signals. Normally, TDOAs of spoofing signals from a common spoofer are identical while those of authentic signals from diverse directions are dispersed. The TDOA is measured as the differential pseudorange to carrier frequency ratio (DPF). In a spoofing case, the DPFs include those of both authentic and spoofing signals, among which the DPFs of authentic are dispersed while those of spoofing are almost overlapped. An algorithm is proposed to search for the DPFs that are within a pre-defined small range, and an alarm will be raised if several DPFs are found within such range. The proposed SNM methodology is validated by simulations and a partial field trial. Results show 99.99% detection and 0.01% false alarm probabilities are achieved. The SNM has the potential to be adopted in various applications such as (1) alerting dedicated users when spoofing is occurring, which could significantly shorten the receiver side spoofing cost; (2) in combination with GNSS performance monitoring systems, such as the Continuous Operating Reference System (CORS) and GNSS Availability, Accuracy, Reliability anD Integrity Assessment for Timing and Navigation (GAARDIAN) System, to provide more reliable monitoring services.

## 1. Introduction

The GNSS’s vulnerability to spoofing was first officially identified by the U.S. government in 2001 [1]. Recently, the situation has become more critical. An experiment conducted by the research team led by Dr. Humphreys illustrates the threat of spoofing, where an unmanned aerial vehicle (UAV) was captured and then forced to crash down [2]. 

Although many effective spoofing detection techniques have been developed to protect individual receivers, there is little discussion in the literature focusing on spoofing monitoring that aims to monitor the presence of spoofing within an area. Actually, spoofing monitoring could be promising and valuable in various applications. For instance, when equipped with spoofing monitoring, one could alert dedicated users when spoofing is occurring. This is attractive because the users can be made aware of spoofing without having to continuously perform spoofing detection themselves. Also, it could be combined with the GNSS performance monitoring systems to provide more reliable services. Nowadays, many systems have been developed for GNSS performance monitoring, such as CORS and GAARDIAN [3]. However, few of them are reported to be equipped with spoofing monitoring techniques. Unfortunately, a performance monitoring service may not be reliable without taking spoofing into consideration. One may doubt the necessity of spoofing monitoring because these systems are equipped with the receiver autonomous integrity monitoring (RAIM) technique, which can detect counterfeit signals, and they tend to have accurate knowledge of their three-dimensional positions so that they can easily detect spoofing based on unexpected position, velocity and time (PVT) outputs. However, this is not always the case. In a spoofing scenario, there are three cases for the status of the monitoring system: (1) The system adopts only the spoofing signals for PVT so that it is spoofed; (2) The system adopts only the authentic signals for PVT, and therefore it is not spoofed and its reported position is not affected by the spoofing. This is likely to happen. For examples, a spoofer is placed somewhat far away from the system so that the received spoofing strength may be weaker than the authentic one. In this scenario, the receiver tends to adopt stronger authentic signals for PVT and ignore the weaker spoofing. In addition, some spoofing technologies are able to spoof only a particular receiver [4]. In this scenario, although other receivers can still receive spoofing signals, their reported PVT will not be influenced; (3) The system adopts a combination of spoofing and authentic signals for PVT. To detect the presence of spoofing, the monitoring mechanism is required to be effective in all three cases. However, the RAIM and the prior known PVT information are not sufficient to satisfy such a requirement. The RAIM cannot be workable for the first two cases because either authentic or spoofing signals tend to be self-consistent with a small pseudorange residual [5], while the prior known PVT information cannot be applied in the second case as the system’s reported PVT is not affected by spoofing. 

Hence, in order to perform spoofing monitoring, one is required to continue detecting spoofing even when not affected by spoofing. Among the proposed spoofing detection techniques, several methods could satisfy such a requirement: (1) Cryptographic based methods [6,7,8] meant to make parts of civil GNSS codes or navigation messages unpredictable to a spoofer. Based on such methods, one can easily find non-authentic signals. Though effective, this requires a modification to the signal structure and may not be available in the near future; (2) The moving receiver-based techniques given by [9,10,11,12]. They assume spoofing signals are transmitted from a common spoofer so that the spoofing parameters, such as signal strengths and Dopplers, are highly correlated. The spoofing detection is developed based on searching for the correlated signals among all the received signals. These techniques are effective, but they require the motion of the receivers, while spoofing monitoring systems/networks tend to be stationary; (3) Redundant signal detection based methods introduced by [4,13,14,15,16] aiming to detect unexpected ‘GNSS-like’ signals. Such methods can be easy to implement as they are software-defined, but they may have trouble distinguishing spoofing from multipath [14,17]. 

Although the aforementioned methods can be effective in spoofing monitoring, they are either difficult to implement or unsuitable for monitoring a network that consists of multiple static receivers. Several anti-spoofing methods based on multiple receivers were proposed in [18,19,20,21,22]. These works assume the multiple receivers adopt only the spoofing signals for PVT and therefore that they will give nearly identical position solutions and pseudorange measurements. Some works [18,19,20] aim to check whether the position solutions reported from multiple spatially separated receivers match their known physical formation; [21] aims to check whether the reported position solutions are almost identical. One study [22] takes advantage of different pseudorange properties between spoofing and non-spoofing cases. In a spoofing case, the pseudoranges of each satellite signal observed at multiple receivers are almost identical, while in a non-spoofing case they are varied. Although these methods are promising as they are effective and can be easily implemented, they can hardly be applied for spoofing monitoring purposes. As discussed before, the PVT solutions from monitoring receivers may not be influenced in the spoofing case and therefore their reported position and pseudorange measurements might not be ‘almost identical’. 

Based on these considerations, a spoofing network monitoring (SNM) mechanism consisting of multiple (more than one) receivers and a central processing component is proposed in this paper. As opposed to the previous works on multi-receiver-based anti-spoofing [18,19,20,21,22], the SNM is able to detect the presence of spoofing signals no matter whether the PVT solutions are influenced by spoofing. The essence of the SNM is to search for the spoofing signals among all the received signals. The use of multiple receivers rather than one receiver is mainly because the SNM mechanism is based on the differing TDOA properties between spoofing and authentic signals. Like many spoofing detection techniques [9,10,11,12,17,18,19,20,22,23], the SNM assumes that the spoofing signals are transmitted from a common spoofer. Hence, the TDOAs of spoofing signals transmitted from a common spoofer are identical, while those of authentic signals from diverse directions are dispersed. The TDOA is measured as the differential pseudorange to carrier frequency ratio (DPF). In a non-spoofing case where only authentic signals are received, the DPFs of the received signals are dispersed. In a spoofing case, both authentic and spoofing signals are received and therefore the DPFs will include those of both authentic and spoofing signals. Among these, the DPFs of authentic are dispersed while those of spoofing are almost overlapped. Hence, an algorithm is designed to search for the DPFs that are within a predefined small range, and an alarm will be raised if several DPFs are found within such a range. Simulations and real-data experiments are conducted to validate spoofing monitoring performance. Results show the detection and false alarm probabilities could reach 99.99% and 0.01%, respectively. 

The SNM is relatively low-cost and can be easily implemented and deployed for three reasons: (1) It consists of at least two monitoring receivers, and most commercial receivers could be adopted with only a slightly modification in software; (2) The monitoring receivers do not require either clock synchronization or the knowledge of their 3-D positions. This implies the SNM could be a ‘plug and play’ option; (3) The methodology is totally software-defined and is of low computational complexity.

The rest of the paper consists of 8 sections. Section 2 gives the architecture of the SNM. Section 3 gives differential pseudorange (DP) models, based on which the DPF models are given in Section 4. Section 5 introduces the spoofing monitoring methodology. Its performance is then tested based on simulations in Section 6. The real-data experiments are conducted in Section 7 to validate the proposed SNM. Section 8 provides some discussion and recommends future work. Section 9 concludes the paper. 

## 2. Spoofing Network Monitoring Architecture

**Figure 1:**
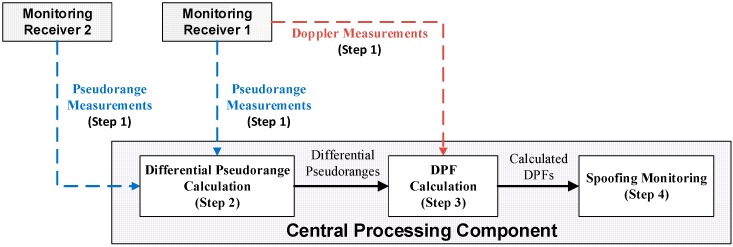
Spoofing network monitoring architecture.

The architecture given by Figure 1 consists of at least two monitoring receivers and a central processing component (CPC). The monitoring receivers are used to provide pseudorange and Doppler measurements of all the received signals. These measurements are then fed into the CPC for spoofing monitoring purposes. The rest of this section introduces the 4-step spoofing network monitoring architecture, and a detailed analysis is given in the successive sections.

### 2.1. Raw Measurements

**Figure 2:**
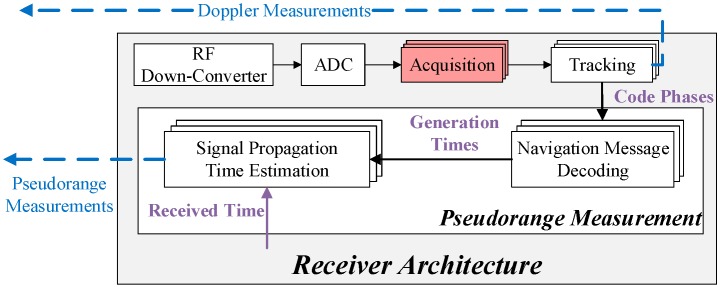
Monitoring Receiver Architecture.

Since the spoofing monitoring is based on the DPF, the first step is to estimate the pseudorange and Doppler measurements of all the received signals. This step is performed by the monitoring receivers. The receiver architecture given by Figure 2 is almost the same as the normal commercial receivers except for the acquisition block. The modified acquisition block searches for all the signals and then passes all those that are above the predetermined acquisition threshold to the tracking block. Hence, if spoofing exists, both the authentic and spoofing signals will be acquired [10,11,12]. This modified acquisition process has already been introduced and adopted by references [10,11,12] for spoofing detection purposes. The tracking block aims to estimate the Doppler and code phase measurements of these acquired signals. The Doppler measurements are fed into the CPC and the code phases are used to measure the pseudorange. It is measured as the product of the speed of light and the signal’s propagation time that is the difference between its generation and received time. The generation time is determined based on the decoded navigation message and the code phase [24] while the received time is read from the receiver’s clock. Though the GNSS cannot be trusted for timing as there may exist a spoofing, many other cheap ways could be adopted. For example, there is the Network Time Protocol (NTP)-based clock synchronization technology, which is widely used for internet time synchronization and can achieve a precision of tens of milliseconds [25]. This precision is sufficient for spoofing monitoring. 

Note the following:

- In a spoofing case, although both authentic and spoofing signals are processed by the receiver, the receiver itself does not know the types of the signals (spoofing or authentic), and it does not even know whether there are spoofing signals or not.
- The monitoring receiver does not need to perform the PVT process because its only function is to provide raw measurements. Hence, the PVT block is not given in the monitoring receiver architecture.

### 2.2. Differential Pseudorange Calculation

The differential pseudorange (DP) is calculated based on the pseudorange measurements from receivers. In a spoofing case, the pseudoranges of both spoofing and authentic signals are provided by each receiver, so the calculated DPs would consist of 2 or 3 DP types: (1) DP between spoofing signals; (2) DP between authentic signals; and possibly (3) the DP between spoofing and authentic signals if the two have some common PRNs. These three types are introduced in Section 3.3, where the corresponding DP models are also given.

### 2.3. DPF Calculation

The DPF is calculated as the ratio between the differential pseudorange and the received carrier frequency. The received carrier frequency is a combination of the signal frequency generated at the satellite (e.g., 1.57542 GHz for GPS L1) and the Doppler measurement provided by either of the two receivers. Based on the DPs given by step 2, the calculated DPFs in a spoofing case also include 2 or 3 types: (1) DPF between spoofing signals; (2) DPF between authentic signals; and possibly (3) the DPF between spoofing and authentic signals.

These three types are discussed in Section 4, where the corresponding DPF models are also given. It shows the DPF consists of the TDOA, multipath error difference, clock difference and estimation noise. Considering spoofing signals are from a common spoofer, the first three parts of spoofing DPFs are identical. Hence, the spoofing DPFs are almost overlapped. By contrast, the authentic DPFs are dispersed as authentic signals come from various directions. 

### 2.4. Spoofing Monitoring

In a non-spoofing case, only dispersed authentic DPFs are present. In a spoofing case, as discussed before, besides dispersed authentic DPFs, the overlapped spoofing DPFs will also be present. Hence the monitoring algorithm is designed to search for the DPFs that are within a predefined small range, and the presence of spoofing is determined when several DPFs are found within such range. The monitoring methodology, including the hypothesis test, the determination of the pre-defined range and the algorithm, is introduced in Section 5.

## 3. Differential Pseudorange Models

This section firstly gives the spoofing scenario, based on which the pseudorange models are then given. Finally, the differential pseudorange models are formulated.

### 3.1. Spoofing Scenario

**Figure 3:**
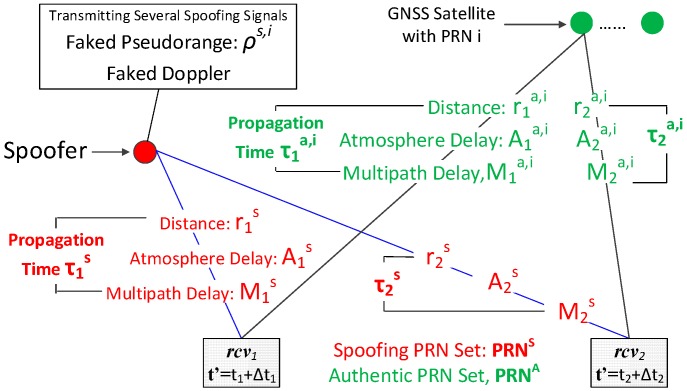
Spoofing Scenario.

Figure 3 illustrates a typical spoofing scenario consisting of two monitoring receivers, a spoofer and several GNSS satellites. It is assumed each of the two monitoring receivers, noted as *rcv*_1_ and *rcv*_2_, are able to receive both spoofing and authentic signals. The clock bias for *rcv_x_* is as ∆*t_x_*. The receiver time *t′* of *rcv_x_* is modelled as a combination of true time *t_x_* and the clock bias ∆*t_x_*:

(1) $$t\prime =t_{x}+\Delta t_{x}$$

The spoofer is transmitting several fake GNSS signals with faked Doppler and code phase, and the faked code phase will result in the faked pseudorange *ρ^s,i^*. Like many other spoofing detection methods assuming all spoofing signals transmitted from a common spoofer, the number of spoofing signals is assumed to be at least 4 so that victim receivers could be spoofed to a wrong location via adopting spoofing signals for PVT calculation. The PRN sets of spoofing and authentic signals are noted as **PRN^S^** and **PRN^A^**, respectively. The relationships between the two sets are as follows:

$$\begin{array}{ccc} PRN^{A\cap S}=PRN^{A}\cap PRN^{S}, & PRN^{A-S}=PRN^{A}-PRN^{S}, & PRN^{S-A}=PRN^{S}-PRN^{A} \end{array}$$

where **PRN^A∩S^** is the intersection of **PRN^A^** and **PRN^S^**, **PRN^A−S^** belongs to **PRN^A^** but not **PRN^S^**, and **PRN^S−A^** belongs to **PRN^S^** but not **PRN^A^**.

### 3.2. Pseudorange Measurement

After all the signals are processed, the pseudorange measurements at *rcv_x_* are:

(2) $${\tilde{\rho }}_{x}=\left[\begin{array}{l} {\tilde{\rho }}_{x}^{a,i},{\tilde{\rho }}_{x}^{s,i}|i\in PRN^{A\cap S}\\ {\tilde{\rho }}_{x}^{a,i}|i\in PRN^{A-S}\\ {\tilde{\rho }}_{x}^{s,i}|i\in PRN^{S-A} \end{array}\right]$$

wherein ${\tilde{\rho }}_{x}^{s,i}$ and ${\tilde{\rho }}_{x}^{a,i}$ are respectively the authentic and spoofing pseudorange measurements at *rcv_x_*. The superscript *i* denotes the PRN index. Note that both ${\tilde{\rho }}_{x}^{s,i}$ and ${\tilde{\rho }}_{x}^{a,i}$ are available for $i\in PRN^{A\cap S}$ because the spoofing and authentic have the common PRN *i*. ${\tilde{\rho }}_{x}^{s,i}$ and ${\tilde{\rho }}_{x}^{a,i}$ measured at receiver time *t′* is derived in Appendix A, and the result is:

(3) $${\tilde{\rho }}_{x}^{s,i}\left(t\prime \right)=\frac{\lambda f^{s,i}}{c}\left[r_{x}^{s}+A_{x}^{s}+M_{x}^{s}+c\Delta t_{x}\right]+\rho^{s,i}\left(t\prime \right)+\xi_{x}^{s,i}$$

(4) $${\tilde{\rho }}_{x}^{a,i}\left(t\prime \right)=\frac{\lambda f^{a,i}}{c}\left[r_{x}^{a,i}\left(t\prime \right)+A_{x}^{a,i}+M_{x}^{a,i}+c\Delta t_{x}\right]+\xi_{x}^{a,i}$$

wherein *λ* is the carrier wavelength and *c* is the speed of light. $f^{s,i}$ ($f^{a,i}$) is the received carrier frequency, which is a combination of the GNSS carrier frequency (e.g., 1.57542 GHz for GPS L1) and Doppler. $r_{x}^{s}$ ($r_{x}^{a,i}$) is the distance between the spoofer (satellite) and *rcv_x_*. *A* denotes the atmosphere induced delay, including troposphere and ionosphere. *M* is the multipath induced error. $\rho^{s,i}\left(t\prime \right)$ is the faked pseudorange measurements simulated at the spoofer at *t′*. The measurement noise $\xi_{x}^{s,i}$ ($\xi_{x}^{a,i}$) is usually modelled as an identical and independently distributed (IID) Gaussian random variable with zero mean and the variance of $\sigma^{2}$ [24]. 

### 3.3. Differential Pseudorange

The differential pseudorange (DP) is calculated as the difference of the pseudorange measurements with the same PRN, which are respectively provided by two receivers. The result is given as below:

(5) $$\Delta \rho =\left[\begin{array}{l} \Delta \rho^{a,i},\Delta \rho^{s,i},\Delta \rho^{a-s,i},\Delta \rho^{s-a,i}|i\in PRN^{A\cap S}\\ \Delta \rho^{a,i}|i\in PRN^{A-S}\\ \Delta \rho^{s,i}|i\in PRN^{S-A} \end{array}\right]$$

where

(6) $$\begin{array}{cccc} \Delta \rho^{s,i}={\tilde{\rho }}_{1}^{s,i}-{\tilde{\rho }}_{2}^{s,i}, & \Delta \rho^{a,i}={\tilde{\rho }}_{1}^{a,i}-{\tilde{\rho }}_{2}^{a,i}, & \Delta \rho^{a-s,i}={\tilde{\rho }}_{1}^{a,i}-{\tilde{\rho }}_{2}^{s,i}, & \Delta \rho^{s-a,i}={\tilde{\rho }}_{1}^{s,i}-{\tilde{\rho }}_{2}^{a,i} \end{array}$$

In a spoofing case, the calculated DPs will include 3 DP types: (1) DP between authentic signals: $\Delta \rho^{a,i}$; (2) DP between spoofing signals $\Delta \rho^{s,i}$; (3) DP between authentic and spoofing signals: $\Delta \rho^{a-s,i}$ and $\Delta \rho^{s-a,i}$. This type exists if and only if $PRN^{A\cap S}$ is a nonempty set. By substituting Equations (3) and (4) into Equation (6), the $\Delta \rho^{s,i}$ and $\Delta \rho^{a,i}$ becomes:

(7) $$\Delta \rho^{s,i}=\lambda f^{s,i}\left(\Delta \tau^{s}+\Delta M^{s}+\Delta t\right)+\Delta \xi^{s,i}$$

(8) $$\Delta \rho^{a,i}=\lambda f^{a,i}\left(\Delta \tau^{a,i}+\Delta M^{a,i}+\Delta t\right)+\Delta \xi^{a,i}$$

where

$$\begin{array}{l} \begin{array}{cccc} \Delta \tau^{s}=\left[r_{1}^{s}-r_{2}^{s}\right]/c, & \Delta M^{s}=M_{1}^{s}-M_{2}^{s}, & \Delta \xi^{s,i}=\xi_{1}^{s,i}-\xi_{2}^{s,i}, & \Delta t=\Delta t_{1}-\Delta t_{2} \end{array}\\ \begin{array}{ccc} \Delta \tau^{a,i}=\left[r_{1}^{a,i}-r_{2}^{a,i}\right]/c, & \Delta M^{a,i}=M_{1}^{a,i}-M_{2}^{a,i}, & \Delta \xi^{a,i}=\xi_{1}^{a,i}-\xi_{2}^{a,i} \end{array} \end{array}$$

In $\Delta \rho^{s,i}$ ($\Delta \rho^{a,i}$), the ∆*τ^s^* (∆*τ^a,i^*) is actually the TDOA of a spoofing signal (authentic signal). The difference of atmosphere delay is negligible for short baseline (e.g., <10 km) differential pseudorange [26], therefore it is neglected in both $\Delta \rho^{s,i}$ and $\Delta \rho^{a,i}$ as the SNM is short baseline based. Also, given that the IID Gaussian distributed measurement noise at different receivers ($\xi_{1}^{s,i}$ and $\xi_{2}^{s,i}$) are uncorrelated [26], the $\Delta \xi^{s,i}$ ($\Delta \xi^{a,i}$) can be modelled as an IID Gaussian random variable with zero mean and the variance of $2\sigma^{2}$ (the standard deviation of the pseudorange measurement noise σ was defined in Section 3.2):

(9) $$\begin{array}{cc} \Delta \xi^{s,i}\sim N\left[0,\sqrt{2}\sigma \right], & \Delta \xi^{a,i}\sim N\left[0,\sqrt{2}\sigma \right] \end{array}$$

wherein **N**[*a,b*] denotes the Gaussian distribution with the mean of *a* and standard deviation of *b*. 

## 4. Differential Pseudorange to Carrier Frequency Ratio

The DPF of each signal is calculated as the ratio between the differential pseudorange and the received carrier frequency. The received carrier frequency measurement is a combination of the standard GNSS frequency and the Doppler measurement. The Doppler can be provided by either of the two receivers. Note that received carrier frequency measurement includes the pure carrier frequency and the Doppler measurement error, and the Doppler measurement error is negligible as it is way smaller than the carrier frequency. Based on the calculated differential pseudoranges given by Equation (5), the calculated DPFs are given as:

(10) $$k=\left[\begin{array}{l} k^{a,i},k^{s,i},k^{a-s,i},k^{s-a,i}|i\in PRN^{A\cap S}\\ k^{a,i}|i\in PRN^{A-S}\\ k^{s,i}|i\in PRN^{S-A} \end{array}\right]$$

where $\begin{array}{cccc} k^{s,i}=\frac{\Delta \rho^{s,i}}{\lambda f^{s,i}}, & k^{a,i}=\frac{\Delta \rho^{a,i}}{\lambda f^{a,i}}, & k^{a-s,i}=\frac{\Delta \rho^{a-s,i}}{\lambda f^{a,i}}, & k^{s-a,i}= \end{array}\frac{\Delta \rho^{s-a,i}}{\lambda f^{s,i}}$.

In a spoofing case, the calculated DPF include three DPF types: (1) DPF between the authentic signals (authentic DPF): *k^a,i^*; (2) DPF between spoofing signals (spoofing DPF): *k^s,i^*; and (3) DPF between authentic and spoofing signals (AS DPF): *k^s-a,i^* and *k^a-s,i^*. By substituting Equations (7) and (8), the *k^s,i^* and *k^a,i^* are given as:

(11) $$k^{s,i}=\underset{\mathrm{Identical}\;\mathrm{Among}\;\mathrm{Spoofing}\;\mathrm{Signals}}{\underset{︸}{\Delta \tau^{s}+\Delta M^{s}+\Delta t}}+\delta^{s,i}$$

(12) $$k^{a,i}=\underset{\mathrm{Various}\;\mathrm{for}\;\mathrm{authentic}\;\mathrm{signals}\;}{\underset{︸}{\Delta \tau^{a,i}+\Delta M^{a,i}}}+\Delta t+\delta^{a,i}$$

where

(13) $$\begin{array}{cc} \delta^{s,i}=\Delta \xi^{s,i}/\left(\lambda f^{s,i}\right), & \delta^{a,i}=\Delta \xi^{a,i}/\left(\lambda f^{a,i}\right) \end{array}$$

The DPF noises $\delta^{s,i}$ and $\delta^{a,i}$ are further derived in Appendix B, and the results are:

(14) $$\begin{array}{ll} \delta^{s,i}=\Delta \xi^{s,i}/c, & \delta^{s,i}\sim N\left[0,\sigma_{\delta }\right]\\ \delta^{a,i}=\Delta \xi^{a,i}/c, & \delta^{a,i}\sim N\left[0,\sigma_{\delta }\right] \end{array}$$

where

(15) $$\sigma_{\delta }=\sqrt{2}\sigma /c$$

As can be seen, both the spoofing and authentic DPFs consist of four parts: TDOA, multipath difference, clock difference and the DPF estimation noise. The first three parts are identical among spoofing DPFs as spoofing signals are transmitted from the common source. Hence, the spoofing DPFs would almost overlay each other within a small range. Note that the spoofing DPFs do not completely overlay due to the random DPF estimation noise. By contrast, the TDOAs and multipath differences of authentic signals are dispersed because they are transmitted from diverse directions. Hence, the authentic DPFs are dispersed. 

Note that the AS DPFs *k^s-a,i^* and *k^a-s,i^* are not given because they are irrelevant for the spoofing monitoring methodology introduced in the following section. This is because in a non-spoofing case, these AS DPFs will not even exist. In a spoofing case, though they might be present, the spoofing monitoring technique is based on the overlapped spoofing DPFs rather than the AS DPFs. 

Following gives the distribution of the *k^s,i^*, which will be used for detection probability analysis given in the following section. Since $\Delta \xi^{s,i}$ given by Equation (9) is an IID Gaussian random variable, the spoofing DPF noise $\delta^{s,i}$ given by Equation (14) is also IID. Hence, the spoofing DPFs *k^s,i^* consisting of identical $\Delta \tau^{s}$, $\Delta M^{s}$ and Δt, and IID Gaussian random variables $\delta^{s,i}$, are identically and independently Gaussian distributed:

(16) $$k^{s,i}∼N\left[\Delta \tau^{s}+\Delta M^{s}+\Delta t,\sigma_{\delta }\right]$$

## 5. Monitoring Methodology

### 5.1. Hypothesis Test

**Figure 4:**
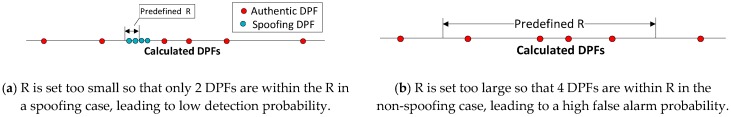
The improper R results in poor monitoring performance.

The spoofing monitoring is based on the calculated DPFs. In a non-spoofing case, the calculated DPFs include only dispersed authentic DPFs. In a spoofing case, however, the calculated DPFs include not only authentic, but also overlapped spoofing DPFs. Furthermore, it is assumed in Section 3.1 that the number of spoofing signals is at least 4. Hence, in a spoofing case, there must present at least 4 DPFs that almost overlay each other but with different PRNs. Hence, the spoofing monitoring is designed to search for the DPFs that are within a predefined small range and the existence of spoofing can be identified if at least 4 DPFs (with different PRNs) are found within such a range. The null hypothesis H_0_ stating the absence of spoofing and alternate hypothesis H_1_ stating the existence of spoofing are given as:

(17) $$\begin{array}{l} H_{0}:\;N\left(R\right)<4\\ H_{1}:\;N\left(R\right)\geq 4 \end{array}$$

wherein *N*(*R*) represents the number of DPFs (with different PRNs) that are within the predefined range of R. A proper R is crucial to the monitoring performance. As is seen in Figure 4, the improper R would result in either low detection or high false alarm probability. Hence, Section 5.2 determines the minimum R required to achieve a desired detection probability. After the R is determined, an algorithm is developed in Section 5.3 to search for the DPFs that are within the R. 

### 5.2. The Lower Bound (LB) on the Detection Probability

Based on the hypothesis test, the detection probability (*P_d_*) is defined as the probability that at least 4 spoofing DPFs are within the predefined range R.

(18) $$P_{d}=\mathrm{Pr}\left\{N\left(R\right)\geq 4\right\}$$

wherein Pr{x} denotes the probability of the event x. Actually, the *P_d_* increases in the number of the received spoofing signals, *m*. This is because more spoofing signals results in more spoofing DPFs. Given more spoofing DPFs, more of them will be within R, leading to the higher *P_d_*. Considering that the *m* cannot be controlled at the monitoring side, this section focuses on the *P_d_* in the worst case, where the *m* achieves the minimum possible value, 4. The *P_d_* in such worst case is denoted as the lower bound (LB) detection probability, ${\tilde{P}}_{d}$

(19) $$P_{d}\geq {\tilde{P}}_{d}=\mathrm{Pr}\left\{N\left(R\right)\geq 4|m=4\right\}=\mathrm{Pr}\left\{N\left(R\right)=4|m=4\right\}$$

The second equality sign is considering the *N*(*R*) cannot be larger than 4 because the *N*(R) always equals or is less than the overall number of DPFs, *m*. Further, given that the 4 spoofing DPFs will be within R if and only if the range of the 4 DPFs is smaller than R, the ${\tilde{P}}_{d}$ is further written as:

(20) $${\tilde{P}}_{d}=\mathrm{Pr}\{\mathrm{maximum}\;\mathrm{DPF}\;\mathrm{Element}-\;\mathrm{minimum}\;\mathrm{DPF}\;\mathrm{Element}\;\leq R\}=\mathrm{Pr}\left\{r\left(4\right)\leq R\right\}$$

wherein *r*(*x*) denotes the range of the *x* spoofing DPFs, which is the difference between the maximum and the minimum DPF element. The cumulative distribution function (cdf) of the *r*(4), *F_r_*_(4)_, is derived in Appendix C and the result is:

(21) $$F_{r(4)}\left(R\right)=\mathrm{Pr}\left\{r(4)\leq R\right\}=4\int_{-\infty }^{\infty }g\prime \left(x\right){\left[G\prime \left(x+R/\sigma_{\delta }\right)-G\prime \left(x\right)\right]}^{3}dx$$

wherein the *g′* and *G′* are respectively the probability density function and the cdf of the zero mean Gaussian with unit variance. Based on Equations (20) and (21), the ${\tilde{P}}_{d}$ can be finally modeled as:

(22) $${\tilde{P}}_{d}=F_{r\left(4\right)}\left(R\right)$$

Based on Equation (22), the minimum R can be also determined based on a required detection probability:

(23) $$R=F_{r(4)}^{-1}\left({\tilde{P}}_{d}\right)$$

**Figure 5:**
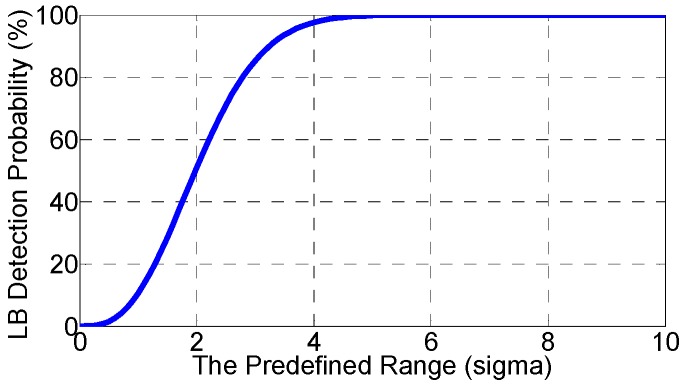
LB detection probability versus the predefined range.

Figure 5 gives the ${\tilde{P}}_{d}$ versus the predefined range R and Table 1 gives the minimum R required to achieve the typical desired detection probabilities. 

### 5.3. Algorithm

The algorithm to deal with the hypothesis test follows the 6 steps:

- Define the range R based on Equation (23).
- Sort the calculated DPFs from minimum to maximum: [*k*_1_ ≤ *k*_2_ ≤ … ≤ *k_n_*].
- Generate a counter *cnt* and initialize it as 1.
- Define a search range [*k_cnt_,k_cnt_ + R*] and calculate the number of elements within the range.
- If the number equals or is over 4, the presence of spoofing is determined. Otherwise, goes to step 6.
- Update *cnt* as: *cnt = cnt +* 1, and goes to step 4 to test the next range.

**Figure 6:**
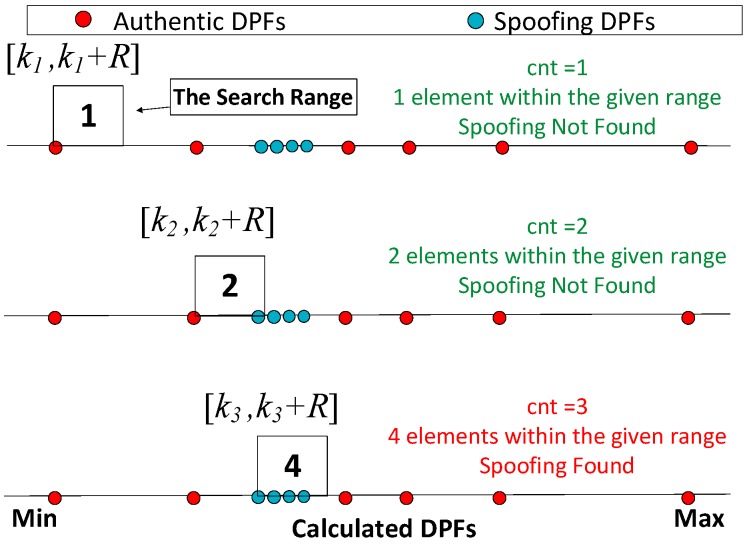
An example to illustrate the spoofing monitoring algorithm.

Figure 6 gives an example to illustrate the algorithm. As the counter *cnt* is 1, only one element (*k*_1_) is in the search range [*k*_1_*,k*_1_ *+ R*], which means the spoofing has not been found. After the *cnt* is updated as 2, 2 elements (*k*_2_ and *k*_3_) are within the search range [*k*_2_*,k*_2_ *+ R*] and therefore the spoofing has not been found. But after the counter is updated as 3, the number of elements within the search range [*k*_3_*,k*_3_ *+ R*] equals to 4. Hence, the alternate hypothesis H_1_ is accepted and the presence of spoofing is identified.

## 6. Performance Analysis

The performance of the SNM can be characterized by the lower bound detection probability (${\tilde{P}}_{d}$) and the false alarm probability (*P_fa_*). The ${\tilde{P}}_{d}$ can be determined by the predefined range R, (see Equation (22)). However, the *P_fa_* has not yet been tested. Hence, Monte Carlo simulations are conducted in this section to test the *P_fa_*. The *P_fa_* is defined as the probability that at least 4 authentic DPFs that are within the predefined range R. 

### 6.1. Simulation Setup

**Figure 7:**
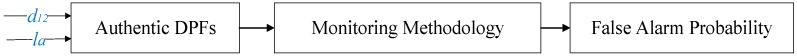
Simulation Setup.

The simulation setup is given by Figure 7. The two inputs are respectively the distance between two receivers (*d*_12_) and the number of authentic DPFs (*l_a_*). Each DPF is generated based on the model given by Equation (12), wherein the multipath difference is sampled from a **N**[0,0.3/*c*]; the clock difference is sampled from a uniform distribution over the range of [−500 ms, 500 ms]; and the DPF estimation noise is sampled from $N\left[0,\sigma_{\delta }\right]$. The $\sigma_{\delta }$ is given by Equation (15), where the pseudorange noise σ is set as typically 0.2 m according to the GPS UERE (user equivalent range error) budget [27]. The TDOA $\Delta \tau^{a,i}$ is generated as:

(24) $$\Delta \tau^{a,i}=H_{i}\Delta x/c$$

where, $H_{i}=\left[-\cos\theta_{i}\sin\alpha_{i},-\cos\theta_{i}\cos\alpha_{i},-\sin\alpha_{i}\right]$, wherein the elevation and azimuth of a GNSS satellite, *θ_i_* and *a_i_*, are randomly sampled over the range of respectively [0,π/2] and [0,2π]. The orientation vector between two receivers **∆x** is sampled from a uniform distribution on the unit sphere. 

### 6.2. Result

**Figure 8:**
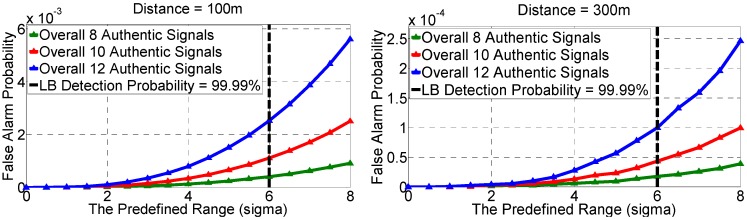
The predefined range versus false alarm probability.

Figure 8 shows the predefined range R versus *P_fa_* for various *d*_12_ and number of authentic signals *l_a_*. As shown, for a given predefined range R, the farther the two receivers are placed, the lower the *P_fa_* would be. This is because the TDOAs of authentic signals will be more dispersed with a longer *d*_12_, resulting in the decrease of the *P_fa_*. On the other hand, a larger number of authentic signals leads to a higher *P_fa_*. This is because more authentic signals give more DPFs, and given more DPFs, it will be more likely that at least 4 of them are within the R, resulting in a higher *P_fa_*. Also, it is found in Section 5.2 that the predefined range of $6\sigma_{\delta }$ could give a ${\tilde{P}}_{d}$ of 99.99%. Fortunately, the *P_fa_* is also satisfactory for such a range. As is illustrated in Table 2, with the R of $6\sigma_{\delta }$ (or equivalently, the ${\tilde{P}}_{d}$ of 99.99%), the *P_fa_* is no more than 2.5 × 10^−3^ (for *d*_12_ = 100 m) or 1.0 × 10^−4^ (for *d*_12_ = 300 m). 

**Figure 9:**
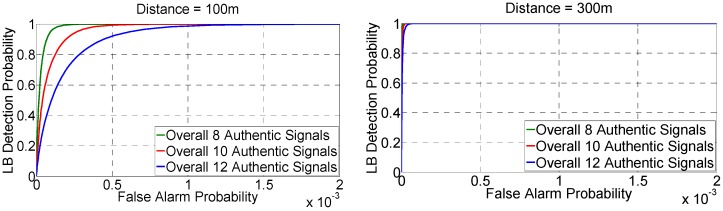
The Receiver Operating Characterization (ROC).

Figure 9 gives the receiver operating characterization (ROC). It shows that the performance is satisfactory especially for the case that the *d*_12_ is set as 300 m. It is noted that the false alarm probability shown by the x-axis ranges from 0 to 2 × 10^−3^ instead of 0 to 1.

## 7. Experiments and Results

### 7.1. Setup

#### 7.1.1. Signal Collection

**Figure 10:**
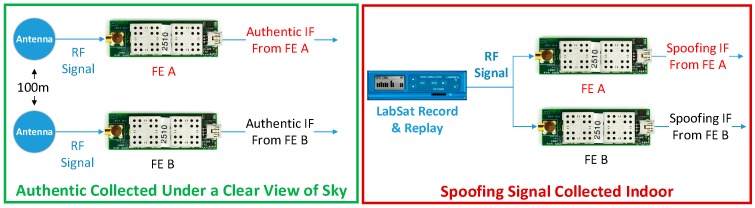
Signal collection: authetic and spoofing signals are collected seperately.

In this experiment, a Labsat GNSS record & replay is adopted as the spoofer. Two GN3SV2 front ends (FE), FE A and B, are used to collect the Intermediate Frequency (IF) GNSS signals. Each FE is connected to a laptop to collect the signals and the local computer time is used as the receiver time. The ideal setup for the data collection is to put the spoofer and the two front ends outside under a clear view of sky, so that both the spoofing and authentic signals can be collected simultaneously. However, it is challenging to conduct such an experiment as it is illegal to transmit spoofing signals outdoors. Hence, the authentic and spoofing signals are collected separately in this experiment. As is shown in Figure 10, the spoofing signals are collected indoors while the authentic signals are collected under a clear view of the sky, and the distance between the two antennas is 100 m. The PRN set of the collected spoofing and authentic signals are given by Table 3. It shows there are three common elements (14, 25 and 32) between the two sets.

#### 7.1.2. Signal Process

**Figure 11:**
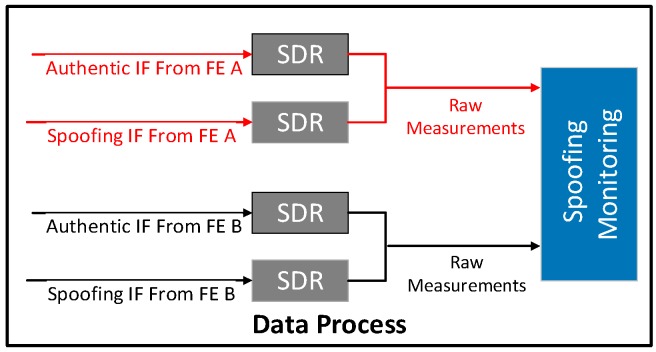
Signal process for spoofing monitoring.

Figure 11 illustrates the workflow of the signal process. As shown, the authentic and spoofing IF signals are respectively fed into a Matlab based software defined radio receiver (SDR) and the outputs are the pseudorange and Doppler measurements. After that, the raw measurements of authentic and spoofing signals from the same FE are combined and are then fed into a spoofing monitoring block. 

#### 7.1.3. The Limitations of the Adopted Experiment

**Figure 12:**
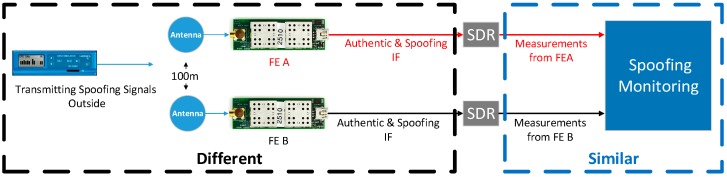
The ideal experimental setup.

The ‘ideal’ experiment discussed before can be expressed by Figure 12, where the spoofer is placed outside under a clear view of sky. By this, the spoofing and authentic signals can be collected and processed by the SDR simultaneously. However, considering conducting such experiment is challenging, the spoofing and authentic IF signals are actually collected and processed separately. The limitation of the adopted experiment is that the modified acquisition process introduced in Section 2 cannot be verified, which is designed to acquire all the signals (spoofing and authentic) simultaneously. Fortunately, such an acquisition process has already been introduced and verified in [10,11,12]. Besides this limitation, the adopted and the ideal experiment setups are similar. As shown, in both cases, the measurements fed into the spoofing monitoring block include both spoofing and authentic. Hence, the verification of the spoofing monitoring technique will not be influenced. 

### 7.2. Result 

#### 7.2.1. DPF

**Figure 13:**
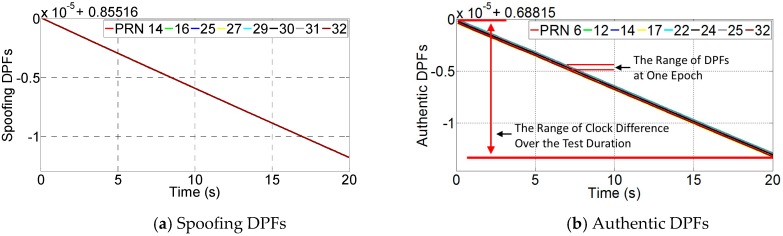
The DPFs over the test duration.

The spoofing monitoring block firstly calculates the DPFs based on the raw measurements. At each epoch, the calculated DPFs include authentic, spoofing and AS DPFs. The spoofing and authentic DPFs are given by Figure 13. It shows all the DPFs decrease over time. This is because each DPF includes clock difference Δt, and it decreases over time due to the clock drift difference. It also shows the spoofing DPFs overlay each other. Note that although the authentic DPFs are actually much more dispersed, it is not obviously illustrated in the figure. This is because the range of the clock difference over the test duration is too large (around 1.5 × 10^−5^ s) compared with the range of the authentic DPFs (from −*d*/*c* to *d*/*c*, where *d* is the distance between two antennas and *c* is the speed of light). Hence, the authentic DPFs seem to be slightly overlapped even though they are not.

**Figure 14:**
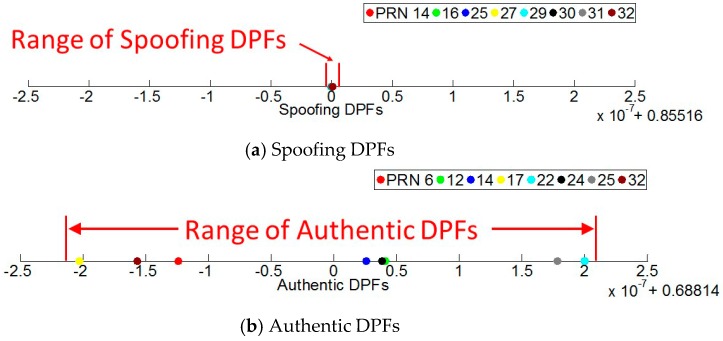
The DPFs for one epoch.

**Figure 15:**
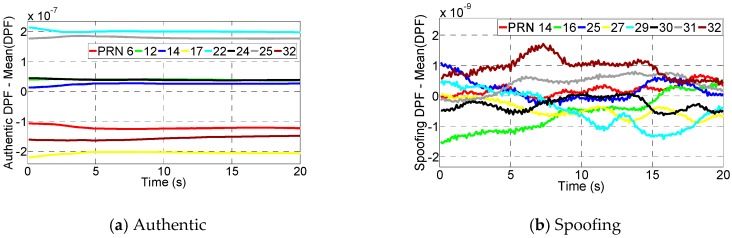
The DPFs are subtracted by their mean value in order to show the DPF range clearly.

In order to illustrate the authentic and spoofing DPF range clearly, Figure 14 gives the DPFs at one epoch (corresponding to the 10th second). As seen, the spoofing DPFs given by Figure 14a are overlapped while authentic DPFs given by Figure 14b are much more dispersed. Further, considering the large range of Δt prevents the range of DPFs from being clearly illustrated, the spoofing and authentic DPFs at each epoch are respectively subtracted by their mean values. By this, the clock difference can be removed and the DPF range over the test duration can be clearly illustrated. Note that this subtraction process only aims to show the range clearly. This process does not affect the range of the DPFs because the DPFs at each epoch are subtracted by a common mean value and therefore their range will remain the same. The results are shown in Figure 15. It shows the authentic DPFs are dispersed over a range of approximately 4 × 10^−7^ while the spoofing DPFs are almost overlapped and are within a range of 3 × 10^−9^.

#### 7.2.2. Spoofing Monitoring

**Figure 16:**
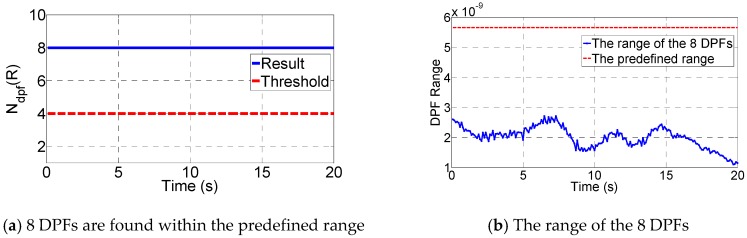
The monitoring result: spoofing is detected because more than 4 DPFs are found within the predefined range.

After the DPFs are calculated, the algorithm introduced in Section 5.3 is performed to test whether there are at least 4 DPFs being within the predefined range that is set as $6\sigma_{\delta }$. The $\sigma_{\delta }$ is calculated based on Equation (15), where the pseudorange noise is set as typically 0.2 m according to the UERE budget [27]. The results are given by Figure 16. As shown in Figure 16a, the number of DPFs that are within the predefined range at every epoch, 8, is larger than the threshold of 4. Hence, the existence of spoofing is determined. Figure 16b further gives the range of the 8 DPFs. It shows the range of the 8 DPFs is much smaller than the predefined range.

Additionally, the monitoring methodology has also been tested in the non-spoofing case, where the spoofing measurements are removed and only the authentic measurements are fed into the spoofing monitoring block. The result shows that only 2 DPFs are found within the predefined range, which correspond to the authentic signals with PRN 12 and 24. 

## 8. Discussions and Future Work

The SNM mechanism proposed in the paper are mainly based on two assumptions: (1) multiple spoofing signals are transmitted from a common spoofer/antenna; and (2) there are 4 or more spoofing signals present in the spoofing case. As is validated, the SNM can be effective under the two assumptions. However, the practicalities of the two assumptions are not clearly given in the previous discussions. Hence, this section firstly discusses the rationality of the two assumptions, based on which our future work is recommended.

### 8.1. The Rationality of the Two Assumptions

#### 8.1.1. Assumption 1: Multiple Fake Signals are Transmitted from a Common Antenna

The proposed SNM mechanism is effective in defending against a single spoofing attack in which multiple spoofing signals are transmitted from a common antenna. Compared with single spoofing, a more advanced spoofing mode is multiple spoofing, which consists of multiple spatially distributed spoofing devices, with each device transmitting a single spoofing signal. In the case of multiple spoofing, however, the TDOA of spoofing signals from various directions will not be overlapped and therefore distinguishing spoofing from authentic signals based on their different TDOA properties becomes impractical, leading to the failure of the proposed SNM mechanism.

Although multiple spoofing could defeat the SNM, such a mode of spoofing is deemed impractical because performing this attack is very challenging [11,13]. In order to defeat the RAIM technique that is currently equipped in most commercial-off-the-shelf (COTS) receivers, the fake pseudoranges (or equivalently the code phases) at the target receiver (s) have to be self-consistent to guarantee small pseudorange residuals. To achieve this, the following three strict requirements need to be satisfied: (1) the clocks of the spatially distributed spoofers need to be synchronized; (2) the processing delay within each spoofing device should be precisely estimated to achieve nano-second level; (3) the spoofing devices should have sub-meter-level knowledge of the three-dimensional position of the target’s antenna phase center. This is almost impossible in the case of moving victim targets. In addition to these three requirements, there are also limitations regarding the placement of the multiple spoofing devices, the cost of the spoofing infrastructure, and the expertise of developing and performing such a spoofing attack.

#### 8.1.2. Assumption 2: There are 4 or More Spoofing Signals Present in the Spoofing Case

The proposed SNM mechanism assumes that there are 4 or more spoofing signals present in the spoofing case, and the presence of spoofing is determined when 4 or more DPFs are found within a predefined small range (or equivalently, overlapped). However, in the case that only a few (less than 4) spoofing signals are present, there will be less than 4 DPFs being overlapped and therefore the SNM mechanism will be failed.

Fortunately, the assumption that there are 4 or more spoofing signals is practical considering the following: if only a few spoofing signals are present (e.g., less than 4), the victim receivers tend to use a combination of spoofing and authentic signals to report PVT solutions. This will lead to the inconsistency among the observed pseudoranges, which can be easily detected by the RAIM [28]. Furthermore, it is not clear how sophisticated the spoofer should be to spoof only a few signals without being detected by RAIM [28]. Although the spoofing might be successful in an extreme scenario, where the signals in view are too few (no more than 4) so that the RAIM is not available (e.g., there are only 4 authentic signals in view and a subset of these signals are spoofed), this scenario is rare and cannot be controlled at the spoofer side. The scenario is highly unlikely for static victims. This is because for static applications such as smart grids, high precision surveys, etc., the receivers are usually placed under a clear view of sky where there are normally sufficient signals (e.g., >10) in view. In terms of moving victims, this scenario is possible, but it is rare and hard to control. For instance, if the targets are moving targets in urban canyons, there might be sometimes no more than 4 visible signals. However, the number of visible signals for moving targets in urban canyon tends to change rapidly. At a certain epoch there are 4 signals in view, but at the next epoch there might be more. Once more than 4 signals are visible, the RAIM becomes available and the spoofing can be detected. And what’s worse, the number of visible signals cannot be controlled at spoofing side.

Hence, in order to defeat the RAIM that is equipped in most current COTS receivers, most spoofers tend to transmit 4 or more spoofing signals and induce the target receivers to use only the spoofing signals for PVT calculation. 

### 8.2. Future Work

Although multiple spoofing is deemed impractical at present [11,13], it might become a threat in the future. In order to be more robust against a spoofing attack, further improvement of the SNM should be focused on defending against multiple spoofing attacks. 

## 9. Conclusions

The proposed SNM is based on the differential pseudorange to carrier frequency ratio (DPF), which is mathematically formulated and analyzed in this paper. As shown, the spoofing DPFs are almost overlapped while authentic DPFs are dispersed. Considering both the overlapped spoofing and dispersed authentic DPFs will be present in the spoofing case, the SNM is designed to search for the DPFs that are within the predefined small range. The predefined range could be determined based on the desired detection probability. This shows that a detection probability of 99.99% can be achieved with the predefined range of $6\sigma_{\delta }$ ($\sigma_{\delta }$ is the DPF estimation noise). Also, false alarm probabilities are tested based on Monte Carlo simulations. As shown, both the predefined range and the distance between two receivers have great impact on false alarm probabilities. With the predefined range of $6\sigma_{\delta }$ and the distance of 300 m, a false alarm rate of 0.01% can be achieved. The effectiveness of the spoofing monitoring technique is validated by real data experiments. It shows that the spoofing DPFs are within a much smaller range than the authentic ones, and overall 8 DPFs are found within the predefined range. This implies the existence of spoofing. 

## Figures and Tables

**Table 1:** The minimum R required to achieve the typical detection probabilities.

LB Detection Probability	99.00%	99.90%	99.99%
Minimum R	$4.4\sigma_{\delta }$	$5.3\sigma_{\delta }$	$6\sigma_{\delta }$

**Table 2:** Numerical P_fa_ for $R=6\sigma_{\delta }$ (${\tilde{P}}_{d}$ = 99.99%).

	Number of Authentic Signals = 8	10	12
**Distance = 100 m**	4.0 × 10^−4^	1.1 × 10^−3^	2.5 × 10^−3^
**Distance = 300 m**	1.8 × 10^−5^	4.3 × 10^−5^	1.0 × 10^−4^

**Table 3:** The spoofing and authentic PRN sets.

Spoofing:	14,16,25,27,29,30,31,32
Authentic:	06,12,14,17,22,24,25,32

## Appendix A

### A.1. Spoofing Pseudorange

As shown in the spoofing scenario given by Figure 1, the spoofing pseudorange measurement at *rcv_x_* at receiver time *t′*, ${\tilde{\rho }}_{x}^{s,i}$, can be modelled as:

(A1) $${\tilde{\rho }}_{x}^{s,i}\left(t\prime \right)=r_{x}^{s}+A_{x}^{s}+M_{x}^{s}+\rho_{x}^{s,i}+c\Delta t_{x}+\xi_{x}^{s,i}$$

wherein the superscript *i* denotes the PRN index and *c* is the speed of light. The pseudorange measurement consists of 6 parts: (1) distance between spoofer and *rcv_x_*: $r_{x}^{s}$; (2) atmosphere delay: $A_{x}^{s}$; (3) multipath delay: $M_{x}^{s}$; (4) the faked pseudorange at *rcv_x_*: $\rho_{x}^{s,i}$; (5) clock Bias: *c*∆*t_x_*; (6) measurement noise: $\xi_{x}^{s,i}$. The first three parts are altogether considered to be the transmission delay between spoofer and *rcv_x_*, $c\tau_{x}^{s}$.

(A2) $$c\tau_{x}^{s}=r_{x}^{s}+A_{x}^{s}+M_{x}^{s}$$

Considering that the $\rho_{x}^{s,i}$ at *rcv_x_* at *t′* is actually that generated at $t\prime -\Delta t_{x}-\tau_{x}^{s}$, it can be further modeled as:

(A3) $$\begin{array}{c} \rho_{x}^{s,i}\left(t\prime \right)=\rho^{s,i}\left(t\prime -\Delta t_{x}-\tau_{x}^{s}\right)=\rho^{s,i}\left(t\prime \right)+\left(\Delta t_{x}+\tau_{x}^{s}\right){\dot{\rho }}^{s,i}\\ =\rho^{s,i}\left(t\prime \right)+\left(\Delta t_{x}+\tau_{x}^{s}\right)\Delta f^{s,i}\lambda \end{array}$$

in which ${\dot{\rho }}^{s,i}$ is the pseudorange rate, which is a product of the faked Doppler $\Delta f^{s,i}$ and GNSS carrier wavelength *λ*. By substituting Equations (A2) and (A3) into Equation (A1), the spoofing pseudorange estimation model becomes:

(A4) $$\begin{array}{cc} {\tilde{\rho }}_{x}^{s,i}\left(t\prime \right) & =\left(c+\Delta f^{s,i}\lambda \right)\left(\tau_{x}^{s}+\Delta t_{x}\right)+\rho^{s,i}\left(t\prime \right)+\xi_{x}^{s,i}\\ & =\frac{1}{c}\left(c+\Delta f^{s,i}\lambda \right)\left[r_{x}^{s}+A_{x}^{s}+M_{x}^{s}+c\Delta t_{x}\right]+\rho^{s,i}\left(t\prime \right)+\xi_{x}^{s,i} \end{array}$$

Further, the *c* can be modelled as a product of *λ* and the GNSS carrier frequency *f* (e.g., 1.57542 GHz for GPS L1 signal): *c= λ f*. Hence, the Equation (A4) can be modified as:

(A5) $${\tilde{\rho }}_{x}^{s,i}\left(t\prime \right)=\frac{1}{c}\lambda f^{s,i}\left(r_{x}^{s}+A_{x}^{s}+M_{x}^{s}+c\Delta t_{x}\right)+\rho^{s,i}\left(t\prime \right)+\xi_{x}^{s,i}$$

where

(A6) $$f^{s,i}=f+\Delta f^{s,i}$$

### A.2. Authentic Pseudorange

The authentic pseudorange measurement at *rcv_x_* at receiver time *t′*, ${\tilde{\rho }}_{x}^{a,i}$ can be modelled as:

(A7) $${\tilde{\rho }}_{x}^{a,i}\left(t\prime \right)=r_{x}^{a,i}+A_{x}^{a,i}+M_{x}^{a,i}+c\Delta t_{x}+\xi_{x}^{a,i}$$

The ${\tilde{\rho }}_{x}^{a,i}$ consists of 5 parts: (1) distance between satellite and *rcv_x_*: $r_{x}^{a,i}$; (2) atmosphere delay, $A_{x}^{a,i}$; (3) multipath delay: $M_{x}^{a,i}$; (4) clock bias: *c*∆*t_x_*; (5) measurement noise: $\xi_{x}^{a,i}$. The first three parts are altogether considered as the transmission delay between satellite *i* and *rcv_x_*, $c\tau_{x}^{a,i}$:

(A8) $$c\tau_{x}^{a,i}=r_{x}^{a,i}+A_{x}^{a,i}+M_{x}^{a,i}$$

Considering the authentic signal received at receiver time *t′*, is actually that generated at the time of $t\prime -\Delta t_{x}-\tau_{x}^{a,i}$, the $r_{x}^{a,i}$ is actually the distance between satellite and *rcv_x_* at time $t\prime -\Delta t_{x}-\tau_{x}^{a,i}$:

(A9) $$\begin{array}{cc} r_{x}^{a,i} & =r_{x}^{a,i}\left(t\prime -\Delta t_{x}-\tau_{x}^{a,i}\right)=r_{x}^{a,i}\left(t\prime \right)+\left(\Delta t_{x}+\tau_{x}^{a,i}\right)v^{a,i}\\ & =r_{x}^{a,i}\left(t\prime \right)+\left(\Delta t_{x}+\tau_{x}^{a,i}\right)\Delta f^{a,i}\lambda \end{array}$$

in which the $v^{a,i}$ is the relative velocity between the satellite and *rcv_x_*, which is a product of carrier Doppler $\Delta f^{a,i}$ and *λ*. By combining Equations (A8) and (A9), $c\tau_{x}^{a,i}$ is further deducted as:

(A10) $$c\tau_{x}^{a,i}=\frac{c}{\left(c-\lambda \Delta f^{a,i}\right)}\left[r_{x}^{a,i}\left(t\prime \right)+\Delta t_{x}\Delta f^{a,i}\lambda +A_{x}^{a,i}+M_{x}^{a,i}\right]$$

By combining Equations (A7), (A8) and (A10), ${\tilde{\rho }}_{x}^{a,i}$ becomes:

(A11) $$\begin{array}{ll} {\tilde{\rho }}_{x}^{a,i}\left(t\prime \right) & =\frac{c}{\left(c-\lambda \Delta f^{a,i}\right)}\left[r_{x}^{a,i}\left(t\prime \right)+\Delta t_{x}\Delta f^{a,i}\lambda +A_{x}^{a,i}+M_{x}^{a,i}\right]+c\Delta t_{x}+\xi_{x}^{a,i}\\ & =\frac{c}{\left(c-\lambda \Delta f^{a,i}\right)}\left[r_{x}^{a,i}\left(t\prime \right)+A_{x}^{a,i}+M_{x}^{a,i}+c\Delta t_{x}\right]+\xi_{x}^{a,i}\\ & =\frac{1}{\left(c-{\left(\lambda \Delta f^{a,i}\right)}^{2}/c\right)}\left(c+\lambda \Delta f^{a,i}\right)\left[r_{x}^{a,i}\left(t\prime \right)+A_{x}^{a,i}+M_{x}^{a,i}+c\Delta t_{x}\right]+\xi_{x}^{a,i}\\ & \approx \frac{1}{c}\left(c+\lambda \Delta f^{a,i}\right)\left[r_{x}^{a,i}\left(t\prime \right)+A_{x}^{a,i}+M_{x}^{a,i}+c\Delta t_{x}\right]+\xi_{x}^{a,i} \end{array}$$

wherein considering the maximum received Doppler for a static receiver is 7 kHz, the ${\left(\lambda \Delta f^{a,i}\right)}^{2}/c$ in the denominator is neglected as it is way smaller than *c*. Also, considering *c* = *λf*, the Equation (A11) is rewritten as:

(A12) $${\tilde{\rho }}_{x}^{a,i}\left(t\prime \right)=\frac{1}{c}\lambda f^{a,i}\left[r_{x}^{a,i}\left(t\prime \right)+A_{x}^{a,i}+M_{x}^{a,i}+c\Delta t_{x}\right]+\xi_{x}^{a,i}$$

where,

(A13) $$f^{a,i}=f+\Delta f^{a,i}$$

## Appendix B

By substituting Equation (A6) into Equation (13), $\delta^{s,i}$ becomes:

(B1) $$\delta^{s,i}=\frac{\Delta \xi^{s,i}}{\lambda \left(f+\Delta f^{s,i}\right)}=\frac{\Delta \xi^{s,i}}{\lambda f}\left[\frac{1}{1+\Delta f^{s,i}/f}\right]$$

Considering that the $\Delta f^{s,i}$ (typically −7 k–7 kHz) is much smaller than the *f* (1.57542 GHz), the $\delta^{s,i}$ is further given as:

(B2) $$\delta^{s,i}=\Delta \xi^{s,i}/\left(\lambda f\right)=\Delta \xi^{s,i}/c$$

wherein the second equals sign is considering the product of the GNSS frequency and wavelength equals to the speed of light: *c = λf*. Further, based on the zero mean Gaussian distributed $\Delta \xi^{s,i}$ given by Equation (9), the spoofing DPF noise $\delta^{s,i}$ is modelled as a zero-mean Gaussian distribution. Likewise, the $\delta^{a,i}$ can be modelled as the same way and the results are:

(B3) $$\delta^{s,i}\sim N\left[0,\sigma_{\delta }\right],\delta^{a,i}\sim N\left[0,\sigma_{\delta }\right]$$

where $\sigma_{\delta }=\sqrt{2}\sigma /c$

## Appendix C

The range of *n* random variables, *X*_1_*, X*_2_, *…, X_n_* , is defined as the difference between the maximum and the minimum of these variables [29]: *r_x_ = max*(*X_i_*) *− min*(*X_i_*)*.* For *n* identically and independently distributed variables, the cumulative distribution function (cdf) of the range is given by [29]:

(C1) $$F_{r_{X}(n)}\left(t\right)=\mathrm{Pr}\left\{r_{X}\leq t\right\}=n\int_{-\infty }^{\infty }l\left(x\right){\left[L\left(x+t\right)-L\left(x\right)\right]}^{n-1}dx$$

wherein the *l*(*x*) and *L*(*x*) are respectively the probability density function (pdf) and the cdf of the distribution of *X_i_*. Since it is analyzed in Section 4 that the spoofing DPFs are identically and independently Gaussian distributed, the cdf of the range of the *m* spoofing DPFs, *F_r_*_(*m*)_, can be modelled based on Equation (C1):

(C2) $$F_{r(m)}\left(R\right)=\mathrm{Pr}\left\{r(m)\leq R\right\}=m\int_{-\infty }^{\infty }g\left(x\right){\left[G\left(x+R\right)-G\left(x\right)\right]}^{m-1}dx$$

wherein *r*(*m*) denotes the range of the m spoofing DPFs, the *g* and *G* are the pdf and cdf of the spoofing DPF distribution, which is the Gaussian distribution with the standard deviation of $\sigma_{\delta }$ (see Equation (15)). The *F_r_*_(*m*)_ can be further derived as:

(C3) $$F_{r(m)}\left(R\right)=m\int_{-\infty }^{\infty }g\prime \left(x\right){\left[G\prime \left(x+R/\sigma_{\delta }\right)-G\prime \left(x\right)\right]}^{m-1}dx$$

wherein the *g’* and *G’* are respectively the pdf and cdf of the zero mean Gaussian distribution with unit variance. Based on this model, the cdf of the range for a case in which the number of spoofing DPFs is *m* = 4 can be modelled as:

(C4) $$F_{r(4)}\left(R\right)=4\int_{-\infty }^{\infty }g\prime \left(x\right){\left[G\prime \left(x+R/\sigma_{\delta }\right)-G\prime \left(x\right)\right]}^{3}dx$$
